# Antihypertensive Medikamente sind nicht mit erhöhter Krankheitsschwere bei atopischer Dermatitis im TREATgermany‐Register assoziiert

**DOI:** 10.1111/ddg.15927_g

**Published:** 2026-02-05

**Authors:** Moritz M. Hollstein, Stephan Traidl, Thomas Werfel, Jochen Schmitt, die TREATgermany Studiengruppe

**Affiliations:** ^1^ Klinik für Dermatologie Venerologie und Allergologie Universitätsmedizin Göttingen; ^2^ Klinik für Dermatologie und Allergologie Medizinische Hochschule Hannover; ^3^ Zentrum für evidenzbasierte Medizin Universitätsklinikum Carl Gustav Carus und Medizinische Fakultät Carl Gustav Carus Technische Universität Dresden

**Keywords:** Atopische Dermatitis, Antihypertensiva, Ekzem, Krankheitsschwere, Register, Antihypertensive medication, atopic dermatitis, disease severity, eczema, registry

## EINLEITUNG

Ekzeme können multifaktoriell bedingt sein. Sie sind durch Erythem, Schuppung und Pruritus gekennzeichnet. Die atopische Dermatitis (AD) ist die Hauptursache,^1^ auch wenn einige Fälle schwer zu diagnostizieren sind: Patienten mit intrinsischer AD können einen späten Krankheitsbeginn, eine erhaltene Hautbarrierefunktion und eine Kontaktsensibilisierung gegenüber Metallen aufweisen.^2^ Auch die Einnahme von Medikamenten kann Ekzeme verursachen.^3^ Kürzlich stellten Ye und Kollegen fest, dass die Verschreibung blutdrucksenkender Medikamente die Inzidenz von Ekzemerkrankungen bei Patienten im Alter von 60 Jahren und darüber von 9 pro 1000 Patientenjahre bei Studienbeginn auf 11 bis 12 pro 1000 Patientenjahre erhöhte.^4^ Insbesondere Diuretika (HR: 1,21, 95%‐Konfidenzintervall [KI]: 1,19‐1,24) und Kalziumkanalblocker (HR: 1,16, 95%‐KI: 1,14–1,18) zeigten einen Zusammenhang mit ekzematösen Läsionen. Um den Zusammenhang zwischen blutdrucksenkenden Medikamenten und AD zu untersuchen, analysierten wir Daten aus dem TREATgermany‐Register, einem der größten AD‐Register weltweit.^5^, ^6^

## METHODIK

TREATgermany, initiiert 2016, ist ein Register, das auf einer prospektiven Beobachtungskohortenstudie von Patienten mit mittelschwerer bis schwerer AD basiert (Ethikgenehmigung *EK TUD 118032016*, clinicaltrails.gov‐Registernummer *NCT03057860*). Die Einschlusskriterien, Methoden und Ziele wurden an anderer Stelle bereits veröffentlicht.^6^ Die Daten für Erwachsene (≥ 18 Jahre) wurden mit Stand März 2022 extrahiert. Für die aktuelle Analyse wurden die Daten der Erstuntersuchung verwendet. Diese umfassten demografische Informationen (zum Beispiel Alter, Geschlecht), Medikation und Krankheitsschwere. Die Krankheitsschwere wurde anhand des objektiven *SCORing Atopic Dermatitis* (oSCORAD), des *Eczema Area and Severity Index* (EASI), des *Patient's Global Assessment* (PGA) und des *Patient‐Oriented Eczema Measure* (POEM) bewertet. Der *Dermatology Life Quality Index* (DLQI) wurde ebenfalls erfasst. Die statistischen Analysen wurden mit den Paketen gtsummary (Version 2.0.0) und ggplot2 (3.5.1) in R (4.2.1) durchgeführt. Quantitative Variablen werden als Mittelwert und Standardabweichung (SD) dargestellt.

## ERGEBNISSE

Insgesamt waren 618 Patienten des Registers weiblich (43,6%) und 799 (56,4%) männlich (Daten auf Anfrage erhältlich). Das Alter lag zwischen 18 und 88 Jahren und betrug im Durchschnitt 40,4 Jahre (Median: 28 Jahre, Q1: 28 Jahre, Q3: 52 Jahre; Tabelle 1). Der Krankheitsbeginn lag im Mittel bei 9,3 Jahren. Von 1417 Patienten erhielten 210 (14,8%) blutdrucksenkende Medikamente (Tabelle 1). Von dieser Gesamtzahl waren 150 (10,6%) ≥ 60 Jahre alt. In dieser Untergruppe betrug das Durchschnittsalter 66,3 Jahre und lag zwischen 60 und 88 Jahren (Median: 64 Jahre, Q1: 62 Jahre, Q3: 69 Jahre; Tabelle 1), und 71/150 (47,3%) erhielten blutdrucksenkende Medikamente. In der Erwachsenenkohorte und der Untergruppe ≥ 60 Jahre war die blutdrucksenkende Medikation nicht mit erhöhter ärztlich erhobener Krankheitsschwere (oSCORAD, EASI) oder dem durch den Patienten angegebenen PGA assoziiert (Tabelle 2). In der Gesamtkohorte, nicht jedoch in der Untergruppe, war die antihypertensive Medikation mit signifikant niedrigeren, das heißt günstigeren, DLQI‐ und POEM‐Werten assoziiert (Tabelle 2).

**TABELLE 1:** Patientencharakteristika.

Merkmal		Antihypertensive Medikation	
	Gesamt	Nein	Ja
	n = 1417^1^	n = 1207^1^	n = 210^1^
**BMI [kg/m^2^]**			
Mittelwert (SD)	25,80 (5,13)	25,24 (4,71)	29,05 (6,21)
n	1399	1193	206
Median, Q1, Q3	25, 22, 28	24, 22, 27	28, 25, 32
**Geschlecht**			
Weiblich	613 (43,5%)	551 (45,8%)	62 (30,1%)
Männlich	795 (56,5%)	651 (54,2%)	144 (69,9%)
n	1.408	1.202	206
**Alter [Jahre]**			
Mittelwert (SD)	40,32 (14,58)	37,72 (13,42)	55,27 (11,71)
n	1.416	1.206	210
Median, Q1, Q3	38, 28, 52	35, 26, 48	56, 48, 62
Bereich	18 ‐ 88	18 ‐ 82	25 ‐ 88
**Alter bei AD‐Krankheitsbeginn [Jahre]**			
Mittelwert (SD)	9,31 (16,18)	7,23 (13,08)	21,20 (24,86)
n	1.392	1.185	207
Median, Q1, Q3	2, 0,11	1, 0, 6	9, 0, 40

Subgruppe ≥ 60 Jahre			
	Gesamt n = 150^1^	Nein n = 79^1^	Ja n = 71^1^
**BMI [kg/m^2^]**			
Mittelwert (SD)	26,28 (4,84)	25,31 (4,30)	27,37 (5,20)
n	149	79	70
Median, Q1, Q3	25, 23, 29	24, 23, 27	27, 24, 30
**Geschlecht**			
Weiblich	55 (36,9%) 149 (99)	33 (41,8%) 79 (100)	22 (31,4%) 70 (99)
Männlich	94 (63,1%) 149 (99)	46 (58,2%) 79 (100)	48 (68,6%) 70 (99)
**Alter [Jahre]**			
Mittelwert (SD)	66,31 (6,13)	65,37 (5,06)	67,35 (7,03)
n	150	79	71
Median, Q1, Q3	64, 62, 69	63, 62, 69	65, 62, 72
Bereich	60 ‐ 88	60 ‐ 82	60 ‐ 88
**Alter bei AD‐Krankheitsbeginn [Jahre]**			
Mittelwert (SD)	30,59 (28,14)	24,81 (25,59)	36,94 (29,59)
n	149	78	71
Median, Q1, Q3	30, 1, 58	14, 1, 53	40, 2, 65

*Abk*.: AD, atopische Dermatitis; BMI, *Body‐Mass‐Index*; Q1, erstes Quartil; Q3, drittes Quartil; SD, Standardabweichung

^1^ n (%). Dateneinträge mit fehlenden Angaben zur antihypertensiven Medikation wurden ausgeschlossen.

**TABELLE 2:** Schweregrad der Erkrankung anhand von Patientenangaben und Arzteinschätzungen, differenziert nach der Einnahme blutdrucksenkender Medikamente.

Merkmal		Antihypertensive Medikation		
	Gesamt	Nein	Ja	p‐Wert^2^
	n = 1417^1^	n = 1207^1^	n = 210^1^	
**PGA**				0,087
Klar	33 (2,36%)	27 (2,26%)	6 (2,94%)	
Fast klar	123 (8,79%)	99 (8,28%)	24 (11,8%)	
Leicht	279 (19,9%)	229 (19,2%)	50 (24,5%)	
Mittelgradig	400 (28,6%)	349 (29,2%)	51 (25,0%)	
Schwer	409 (29,2%)	361 (30,2%)	48 (23,5%)	
Sehr schwer	155 (11,1%)	130 (10,9%)	25 (12,3%)	
n	1399	1195	204	
**DLQI**				0,006
Mittelwert (SD)	11,73 (7,83)	11,95 (7,77)	10,47 (8,07)	
n	1.401	1.197	204	
**POEM**				0,001
Mittelwert (SD)	16,63 (7,68)	16,91 (7,61)	14,96 (7,93)	
n	1.399	1.195	204	
**EASI**				0,2
Mittelwert (SD)	15,68 (12,78)	15,79 (12,74)	15,02 (13,04)	
n	1.409	1.201	208	
**oSCORAD**				0,3
Mittelwert (SD)	39,83 (16,55)	40,00 (16,26)	38,90 (18,16)	
n	1.411	1.204	207	

**Subgruppe ≥ 60 Jahre**				
	Gesamt n = 150^1^	Nein n = 79^1^	Ja n = 71^1^	
**PGA**				0,5
Klar	5 (3,38%)	2 (2,53%)	3 (4,35%)	
Kast klar	16 (10,8%)	5 (6,33%)	11 (15,9%)	
Leicht	28 (18,9%)	17 (21,5%)	11 (15,9%)	
Mittelgradig	38 (25,7%)	20 (25,3%)	18 (26,1%)	
Schwer	40 (27,0%)	23 (29,1%)	17 (24,6%)	
Sehr schwer	21 (14,2%)	12 (15,2%)	9 (13,0%)	
n	148	79	69	
**DLQI**				0,9
Mittelwert (SD)	10,18 (7,56)	10,13 (7,32)	10,23 (7,89)	
n	148	79	69	
**POEM**				0,15
Mittelwert (SD)	14,84 (8,37)	15,80 (8,46)	13,75 (8,20)	
n	148	79	69	
**EASI**				0,7
Mittelwert (SD)	16,56 (14,61)	17,36 (15,76)	15,64 (13,22)	
n	148	79	69	
**oSCORAD**				0,9
Mittelwert (SD)	40,50 (17,53)	41,15 (16,65)	39,75 (18,60)	
n	147	79	68	

Abk.: DLQI, dermatology life quality index; EASI, eczema area severity index; oSCORAD, objective scoring atopic dermatitis; PGA, patient global assessment; POEM, patient‐oriented eczema measure; SD, Standardabweichung

^1^ n (%). Dateneinträge mit fehlenden Angaben zur antihypertensiven Medikation wurden ausgeschlossen.

^2^ p‐Werte wurden mit Wilcoxon‐Rangsummentests (DLQI, POEM, EASI, oSCORAD) und dem exakten Test von Fisher (PGA) berechnet.

## DISKUSSION

Vor dem Jahr 2024 deuteten Forschungsarbeiten auf einen möglichen Zusammenhang zwischen der Einnahme von Kalziumkanalblocker und Ekzemen hin (Tabelle S1 im Online‐Supplement). Kürzlich fanden Ye et al. heraus, dass mehrere blutdrucksenkende Medikamente mit erhöhter Ekzeminzidenz bei Patienten ≥ 60 Jahren assoziiert sind: Diuretika, Kalziumkanalblocker, ACE‐Hemmer und β‐Blocker.^4^ Die Effektgröße war für ACE‐Hemmer am geringsten (HR: 1,02, 95%‐KI: 1,00–1,04). Es blieb unklar, ob die Zusammenhänge kausal und klinisch bedeutsam sind.

Von den fünf *Read‐Codes* (Read ist ein System klinischer Codes, das früher von der britischen Gesundheitsbehörde zur Erfassung von Patientendaten in der Primär‐ und Sekundärversorgung verwendet wurde), die von Ye und Kollegen analysiert wurden, können einige direkt mit AD bei Patienten ≥ 60 Jahren in Verbindung gebracht werden: „Atopische Dermatitis/Ekzem“, „Infantiles Ekzem“, „Beugeekzem“ und „Allergisches (intrinsisches) Ekzem“. Statistisch gesehen können auch viele andere, nicht weiter spezifizierte, „Ekzem‐NOS“‐Fälle auf AD zurückgeführt werden: Auf der Grundlage einer Querschnittsanalyse von 203 813 Teilnehmern des Forschungsprogramms *All of Us* waren 51 % der Ekzemfälle bei Jugendlichen auf eine AD zurückzuführen.^1^

Unsere Analysen zeigen, dass die Einnahme blutdrucksenkender Medikamente weder in der gesamten (erwachsenen) AD‐Kohorte noch in der Untergruppe ≥ 60 Jahre mit einer erhöhten Krankheitsschwere verbunden war. In einer anderen Kohorte von Patienten mit Herzinsuffizienz konnte unsere Gruppe ebenfalls zeigen, dass Pruritus, ein Hauptsymptom des Ekzems, nicht mit antihypertensiver Medikation assoziiert war.^7^ Trotz einer viel kleineren Stichprobengröße in beiden Analysen weisen diese Daten auf eine vernachlässigbare klinische Relevanz der antihypertensiven Medikation für die Krankheitsschwere hin. Theoretisch wäre ein kausaler Zusammenhang zwischen Ekzemen und blutdrucksenkenden Medikamenten auch überraschend, wenn man die ungleichen pharmakologischen Ansatzpunkte blutdrucksenkender Medikamente bedenkt. Im Falle der Kalziumkanalblocker wurde ein möglicher (spezifischer) Pathomechanismus vorgeschlagen.^4^ Die Kalziumkanalblocker‐induzierte Eisenaufnahme und ‐speicherung in menschlichen Keratinozyten kann dort Apoptose und Spongiose bedingen. Diese Veränderungen können klinisch und histomorphologisch einem Ekzem entsprechen.

Beim Vergleich der vorgestellten Ergebnisse aus der TREATgermany‐Kohorte mit den Erkenntnissen von Ye et al. sollte Folgendes beachtet werden: Erstens werden bei TREATgermany keine leichten AD‐Fälle erfasst. Zweitens wurde die AD in TREATgermany von Dermatologen diagnostiziert, während Ye et al. die Ekzem‐Read‐Codes aus der Primärversorgung analysierten. Einschränkungen der vorliegenden Analysen sind in der ausschließlichen Verwendung von Ausgangsdaten zu sehen, wodurch möglicherweise Änderungen der Medikation und des Krankheitsstatus im Laufe der Zeit übersehen werden. Darüber hinaus schränken die relativ geringe Anzahl von Patienten in den Untergruppen und das Studiendesign kausale Schlussfolgerungen und die Verallgemeinerbarkeit ein.

Zusammenfassend lässt sich sagen, dass die Einnahme von Antihypertensiva in der TREATgermany‐Kohorte nicht mit erhöhter Krankheitsschwere verbunden war.

## DANKSAGUNG

Die Autoren danken den teilnehmenden Patienten, Ärzten und Klinikmitarbeitern, dem Dokumentationsteam und nicht zuletzt der TREATgermany‐Studiengruppe (wie auf treatgermany.org aufgeführt) für ihre wesentlichen Beiträge zu dieser Arbeit.

## DANKSAGUNG

Open access Veröffentlichung ermöglicht und organisiert durch Projekt DEAL.

## FINANZIERUNG

TREATgermany ist ein akademisches, Prüfer‐initiiertes klinisches Krankheitsregister, das finanziell von AbbVie Deutschland GmbH & Co. KG, Almirall Hermal GmbH, Galderma S.A., LEO Pharma GmbH, Lilly Deutschland GmbH, Pfizer Inc. und Sanofi unterstützt wird.

## INTERESSENKONFLIKT

J.S. berichtet über institutionelle Zuwendungen für prüfungsinitiierte Forschung durch den Gemeinsamen Bundesausschuss, das Bundesministerium für Gesundheit, das Bundesministerium für Forschung, die Europäische Union, das Bundesland Sachsen, Novartis, Sanofi, ALK und Pfizer. Er nahm als bezahlter Berater an Beiratssitzungen für Sanofi, Lilly und ALK teil und ist Mitglied des Deutschen Nationalen Rates für Gesundheit und Pflege im Bundesministerium für Gesundheit. S.T. und M.H. haben keinen Interessenkonflikt angegeben. T.W. hat Honorare für Vorträge oder wissenschaftliche Beratung zur atopischen Dermatitis von AbbVie, Almirall, Galderma, Janssen/JNJ, LEO Pharma, Leti, Lilly, Novartis, Pfizer und Regeneron/Sanofi erhalten.

## Supporting information



Supplementary information
