# Supplementary material for: Antihypertensive Medikamente sind nicht mit erhöhter Krankheitsschwere bei atopischer Dermatitis im TREATgermany‐Register assoziiert
Source: J Dtsch Dermatol Ges. 2026 Feb 5;24(2):252–5. [Article in German] doi: 10.1111/ddg.15927_g (PMC12875156; doi:10.1111/ddg.15927_g)
Supplement: Supplementary file 1 — Supplementary information [file DDG-24-252-s001.docx]

**Supplements**

Die TREATgermany-Studiengruppe wird von den PSs J. Schmitt (Dresden), S. Weidinger (Kiel) und T. Werfel (Hannover) geleitet und besteht aus den in der Autorenliste genannten Rekrutierungszentren sowie den folgenden Rekrutierungszentren: M. Hilgers, Kliniken für Dermatologie und Allergologie, Universitätsklinikum Aachen, Aachen, Deutschland/ M. Bell, Praxis Dr. med. Magnus Bell, Andernach/ M. Worm, Abteilung für Dermatologie, Allergologie und Venerologie, Charité Berlin/ C. Handrick, Praxis Dr. med. Christiane Handrick, Berlin/ T. Schirmer, Praxis Dr. med. Thomas Schirmer, Berlin/ J. Rossbacher, Praxis Jens Rossbacher/ Dr. med. Klaus Spickermann, Hautzentrum, Friedrichshain/ T Bieber, Abteilung für Dermatologie und Allergologie, Universitätsklinikum Bonn/ U Schwichtenberg, Praxen Derma-Nord, Bremen/ K Neubert, Praxis Dipl.-Med. Kathrin Neubert, Burgstaedt/ B Gerlach, Praxis Dr. med. Beatrice Gerlach, Dresden/ U Boashi, Praxis Dr. med. Ute Boashie, Dresden/ B Homey, Abteilung für Dermatologie und Allergologie, Universitätsklinikum Düsseldorf/ M Mempel, Praxis Prof. Dr. med. Martin Mempel, Elmshorn/ M Sticherling, Abteilung für Dermatologie, Universität, Deutsches Zentrum für Immuntherapie, Erlangen/ SH Hong-Weldemann, Praxis Dr. med. Sung-Hei Hong-Weldemann, Freiburg/ E Tchitcherina, Praxis für Haut- und Geschlechtskrankheiten, Friedberg/ P Buck, Praxis Dr. med. Philipp Buck, Goldbek medical, Hamburg/ M Augustin, Institut für Gesundheitsdienstleistungsforschung in der Dermatologie Hamburg, Universitätsklinikum Hamburg-Eppendorf/ M Pawlak, Praxis Dr. med. Anika Huenermund und Mario Pawlak, Heiligenstadt/ T Schaefer, Praxis Dr. med. Thomas Schaefer/ Dr. med. Doreen Belz, Derma Köln, Köln/ B Schwarz, Praxis Dr. med. Beate Schwarz, Langenau/ P Staubach-Renz, Klinik für Dermatologie, Universitätsklinikum Mainz/ T Biedermann, Abteilung für Dermatologie und Allergologie, Medizinische Fakultät, Technische Universität München/ F Schenck, Dermatologisches Zentrum Hannover/ M Stahl, Praxis Dr. med. Maren Stahl, Osterode/ R von Kiedrowski, Fokuspraxis für chronisch-entzündliche Hauterkrankungen, Hautkrebs und Allergologie sowie Studienzentrum CMS3 (Gesellschaft für Medizinische Studien und Dienstleistungen), Selters/Westerwald.

Tabelle S1. Frühere Untersuchungen über Ekzeme und die Einnahme von blutdrucksenkenden Medikamenten.

| Typ | Stich-probe | Medikament | Ergebnis | Ekzem Typ | Referenz |
| --- | --- | --- | --- | --- | --- |
| Retrospektive Studie (explorativ)^1^ | 83 Patienten im Alter von 65+ | Alle | Nachweis eines Zusammenhangs zwischen ekzematöser Hautveränderung und Medikamenteneinnahme | Ekzematöse Hautveränderung | Morin et al. 2002 Ann Derm Venerol |
| Medsafe-Berichte^2^ | Fallberichte aus Neuseeland | CCBs (Amlodipin, Felodipin) | Zwei Fälle von Ekzemen bei Erwachsenen, die mit der Einnahme von CCB in Verbindung gebracht werden; die Daten reichen nicht für endgültige Schlussfolgerungen aus. | Ekzematöse Dermatitis | Medsafe-Bericht 2004 |
| Französische Fall-Kontroll-Studie^3^ | 102 Patienten im Alter von 60 Jahren und älter mit 204 Kontrollen | CCBs | OR 2,50 (95% CI: 1,30-4,60) für ekzematöse Dermatitis nach CCB-Einsatz. | Ekzematöse Dermatitis | Joly et al. 2007 JID |
| U.S. Fall-Kontroll-Studie^4^ | 94 Patienten im Alter von 50 Jahren und älter mit 132 angepassten Kontrollen | CCBs, Thiazide | OR 4,21 (95% CI: 1,77-9,97) für CCBs; OR 2,07 (95% CI: 1,08-3,96) für Thiazide. | Ekzematöser Hautausschlag | Summers et al. 2013 JAMA Derm |
| Kohortenstudie im Vereinigten Königreich^5^ | 1,5 Millionen ältere Erwachsene im Vereinigten Königreich, 60 Jahre und älter | Diuretika, CCBs, ACE-Hemmer | Diuretika (HR 1,21) und CCBs (HR 1,16) hatten das höchste Risiko; ACE-Hemmer (HR 1,02) und Betablocker hatten das niedrigste Risiko. | Ekzematöse Dermatitis | Ye et al. 2024 JAMA Derm |

CCBs = Kalziumkanalblocker, CI = Konfidenzintervall, HR = Hazard Ratio, OR = Odds Ratio

Andere veröffentlichte Daten umfassen eine Übersichtsarbeit^6^ und zwei Fallberichte^7,8^.

Literatur

1. Morin C, Joly P, Courville P, et al*.* [Chronic eczematiform eruption in the elderly]. *Ann Dermatol Venereol.* 2002;129(1 Pt 1):19-22.

2. Calcium channel blockers and the possible risk of new-onset eczema. https://www.medsafe.govt.nz/safety/Alerts/Calcium_channel_blockers_and_possible_risk_of_new_onset_eczema.asp.[ Last accessed 8 Apr, 2025].

3. Joly P, Benoit-Corven C, Baricault S, et al*.* Chronic eczematous eruptions of the elderly are associated with chronic exposure to calcium channel blockers: results from a case-control study. *J Invest Dermatol.* 2007;127:2766-71.

4. Summers EM, Bingham CS, Dahle KW, et al*.* Chronic eczematous eruptions in the aging: further support for an association with exposure to calcium channel blockers. *JAMA Dermatol.* 2013;149:814-8.

5. Ye M, Chan LN, Douglas I, et al*.* Antihypertensive Medications and Eczematous Dermatitis in Older Adults. *JAMA Dermatol.* 2024;160:710.

6. Tétart F, Joly P. Eczema in elderly people. *Eur J Dermatol.* 2020;30:663-7.

7. Yoo J, Jue M-S. Intractable pruritus with chronic eczema in an elderly patient caused by long-term intake of calcium channel blocker. *Contact Dermatitis.* 2017;77:339-40.

8. Abdelwahab R, Tangalos EG, Matulis J. Differentiation of hydrochlorothiazide-induced dermatitis from stasis dermatitis. *BMJ Case Rep.* 2022;15:e249884.6. Tétart F, Joly P. Eczema in elderly people. *Eur J Dermatol.* 2020;30:663-7.
